# Fence Removal Enhances Elephant Movement and Promotes Behavioural, Physiological and Ecological Functioning

**DOI:** 10.1002/ece3.73619

**Published:** 2026-05-12

**Authors:** Brooke Friswold, Antoinette van de Water, Brett Mitchell, Sally House, Jaco Mitchell, Harin Aiyanna, Audrey Delsink, Marion Garaï, Andre Ganswindt, George Gale

**Affiliations:** ^1^ Conservation Ecology Program King Mongkut’s University of Technology Thonburi Bangkok Thailand; ^2^ Bring the Elephant Home Cape Town South Africa; ^3^ School of Agriculture and Science University of KwaZulu‐Natal Pietermaritzburg South Africa; ^4^ Elephant Reintegration Trust Port Alfred South Africa; ^5^ Elephant Conservation and Research Fund San Diego California USA; ^6^ CIRGEO Interdepartmental Research Center of Geomatics University of Padova Legnaro Italy; ^7^ Humane World for Animals Washington DC USA; ^8^ Mammal Research Institute University of Pretoria Hatfield South Africa

**Keywords:** African elephant, behavioural ecology, elephant well‐being, landscape connectivity, stress‐related biomarkers, wildlife management

## Abstract

Fenced reserves are widely used to conserve and manage African savanna elephants ( *Loxodonta africana* ) in South Africa. However, the effects of fencing and fence removal on elephant wellbeing, movement and ecosystem dynamics remain limited in understanding, with few studies incorporating these elements. We hypothesised that fence removal would increase elephant movement flexibility and range, reduce behavioural disturbance and increase sociality, reduce stress‐related biomarkers and redistribute browsing pressure. We evaluated how a resident elephant population in a private reserve in the Eastern Cape, South Africa, responded to the removal of two internal fences by integrating focal behavioural observations, faecal glucocorticoid metabolite (fGCM) analyses, NDVI‐based vegetation assessments and GPS collar data collected before and after fence removal. Fence removal increased home‐range extent, seasonal mobility and inter‐herd spatial overlap, with elephants incorporating newly accessible habitat at variable rates. Initial movement was towards lower density environments during the exploratory phase, with greater distribution in space utilisation over time. Behavioural responses indicated short‐term increases in aggression, vigilance and disturbance‐related behaviours immediately after fence removal, followed by stabilisation, reduced aggression and increased affiliative behaviour over time. Activity budgets shifted towards increased locomotion and reduced foraging, consistent with exploratory behaviour. fGCM concentrations declined significantly following fence removal and remained significant after accounting for demographic and environmental variables, suggesting reduced physiological stress. Vegetation dynamics were spatially heterogeneous yet comparable between areas of elephant presence and absence. This suggests that expanded access redistributed browsing pressure without causing disproportionate vegetation loss with vegetation change primarily driven by environmental variability and not elephant access. These findings suggest that fence removal can promote more natural movement, behavioural responses and physiological regulation, while maintaining vegetation dynamics consistent with background climatic variability. Fence removal may therefore provide an alternative or complementary approach to population management by alleviating spatial constraints and redistributing ecological pressure across the landscape.

## Graphical abstract

We assessed the effects of internal fence removal on elephant movement, behaviour, physiological stress and vegetation dynamics in a fenced reserve in South Africa using GPS tracking, behavioural observations, faecal glucocorticoid analyses and NDVI‐based metrics. Fence removal increased home‐range extent, seasonal mobility and inter‐herd overlap, reduced stress‐related biomarkers and localised browsing pressure and triggered behavioural adjustments consistent with exploratory expansion followed by increased stabilisation and affiliation. Vegetation responses were comparable between areas of elephant presence and absence before and after increased elephant access, indicating redistribution of browsing pressure without sustained degradation. Fence removal therefore represents a management strategy that supports elephant well‐being and movement patterns while maintaining ecosystem resilience.

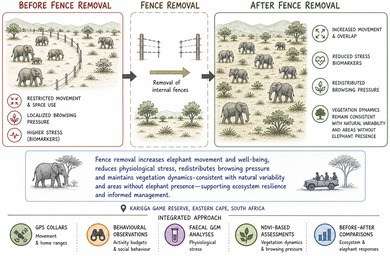

## 1 Introduction

African savanna elephants ( *Loxodonta africana* ) are keystone megaherbivores and ecosystem engineers that shape vegetation structure, biodiversity and seed dispersal across African savanna ecosystems (Bond 1994; Haynes 2012; Pringle 2008; Kerley and Landman 2006; Mapaure and Campbell 2002; Nasseri et al. 2011; Bunney et al. 2017). Listed as Endangered (Gobush et al. 2021), they are also culturally and economically important, particularly through wildlife tourism (Lindsey et al. 2007; van de Water et al. 2022). However, African savanna elephants face increasing threats from habitat loss and fragmentation, poaching, human–elephant conflict and climate change (Chase et al. 2016; Schlossberg et al. 2020). Across much of Africa, elephant populations are declining (Gobush et al. 2021; Edwards et al. 2024), yet southern Africa has seen localised increases due to protection efforts (Selier et al. 2016; Ferreira et al. 2024) with South Africa showing sustained growth (Ferreira et al. 2017; Louw et al. 2021), aided by anti‐poaching efforts, cessation of culling, historical translocations and the establishment of fenced reserves (Slotow et al. 2005; Chase et al. 2016; Schlossberg et al. 2020; Pretorius et al. 2019). However, accelerating habitat fragmentation, including the use of fencing to delineate and protect wildlife areas, can restrict natural movements and increase population isolation, undermining long‐term ecological and demographic stability (Bissonette and Adair 2008; Edwards et al. 2024).

Currently, most elephants in South Africa live in fenced and isolated reserves (Snijders 2014a; Garaï et al. 2022), raising concerns about genetic viability, behavioural stability and long‐term ecological resilience (Pretorius et al. 2019; Huang et al. 2022). This pattern has been strongly shaped by South Africa's private wildlife ownership framework, formalised under the Game Theft Act (Act 105 of 1991), which incentivised fencing to establish legal ownership of wildlife – contributing to population fragmentation (Delsink et al. 2024). While fencing can reduce poaching and enable targeted management (Anthony and Avery 2008; Osipova et al. 2018), it also imposes constraints on wide‐ranging species like elephants by limiting access to critical resources (van Aarde et al. 2006; Harris et al. 2008), intensifying localised browsing and competition (Landman et al. 2008, 2013) and disrupting natural social structures and genetic dispersal (Munshi‐South et al. 2008; Slotow et al. 2005; Whitehouse and Schoeman 2003). This can result in human interventions that may elevate stress‐related biomarkers in elephants (Ganswindt et al. 2003; Viljoen et al. 2008). Fencing also restricts elephants' ability to track spatiotemporal variation in resources (Boone and Hobbs 2004; Shrader et al. 2010; Wato et al. 2016).

Effects of restricted space can be most severe in small, fenced reserves (Novellie et al. 1991), where reduced population size contributes to incomplete social structures, loss of long‐term knowledge, behavioural irregularities, ecological pressure and heightened competition (Pretorius 2004; Slotow et al. 2005; Shannon et al. 2022; Garaï et al. 2023). Fencing shapes vegetation dynamics by concentrating elephant browsing (Loarie et al. 2009), causing woody cover loss, altered plant community composition and localised degradation (Landman et al. 2008; Guldemond and van Aarde 2007). Whereas, increased habitat availability can redistribute browsing pressure and facilitate vegetation recovery (Tucker 1979; Boone and Hobbs 2004; Gandhi et al. 2015). Consequently, fencing may achieve short‐term protection while undermining long‐term ecological integrity and well‐being (Snijders 2014b; Smith et al. 2020; Zungu and Slotow 2022). Fencing thus underscores both the effectiveness of regional conservation measures and the need to better understand how it shapes ecological and well‐being outcomes (Knight and Cowling 2011).

Fence removal, habitat expansion and corridor creation are therefore increasingly recognised as critical conservation strategies (Osborn and Parker 2003), particularly under accelerating climate change (Pretorius et al. 2019; Lindsey et al. 2011). South Africa specifically has designated fence removal and restoring connectivity as key long‐term biodiversity conservation goals for the country (Selier et al. 2025). Therefore, understanding how fence removal affects these ecological processes is critical for assessing long‐term sustainability of reserve‐based conservation (Morrison 2019). In contrast to small, fragmented systems, larger and more connected landscapes reduce the need for intensive management interventions (van de Water et al. 2024; Huang et al. 2024) and promote ecological resilience (van Aarde and Jackson 2007; Giliba et al. 2023; Cook et al. 2025). Restoring landscape connectivity (often via fence removal) can alleviate spatial constraints and promote more natural movement and behavioural patterns in elephants (Osborn and Parker 2003; Druce et al. 2008). Increased habitat availability may also reduce highly localised browsing pressure and human‐elephant conflict by allowing elephants to redistribute across larger areas (Osipova et al. 2018; Pandraud et al. 2020). Expanding habitat availability through fence removal can therefore mitigate both animal well‐being and ecological pressures while supporting conservation objectives (van Aarde and Jackson 2007; Druce et al. 2008; Huang et al. 2024; van de Water et al. 2024).

Despite growing interest, empirical evidence on the ecological, spatial, physiological and behavioural responses to fence removal remains limited and context dependent. As a result, the broader outcomes of restoring connectivity for elephant socioecology, well‐being and ecosystem dynamics remain poorly understood. Most studies have examined individual components of fence removal, including movement patterns (Loarie et al. 2009; Graham et al. 2009), behavioural responses (Szott et al. 2019), physiological stress (Jachowski et al. 2012; Szott et al. 2020a) and vegetation impacts (Guldemond and van Aarde 2007; Owen‐Smith et al. 2019). However, integrated studies combining movement, behaviour, stress‐related biomarkers and ecological metrics remain limited. Such integrated assessments are essential for evidence‐based management, guiding decisions about long‐term reserve design and the future of elephant conservation and management (Fritz 2017). The Eastern Cape of South Africa, where the study was conducted, has high potential for future wildlife corridor expansion under climate‐change projections (Zacarias and Loyola 2018), and has been identified as a priority landscape within South African National Parks' 2040 Mega Living Landscapes vision, including proposed connectivity between Addo Elephant National Park and Mountain Zebra National Park (SANParks 2024), which includes connectivity with private reserves and landowners (Lichtenberg et al. 2025). This study evaluates how internal fence removal in a private reserve influences elephant movement, behaviour, stress‐related biomarkers and vegetation dynamics within a private reserve by integrating GPS tracking, focal behavioural observations, faecal glucocorticoid analyses and NDVI‐based vegetation assessments before and after fence removal.

Specifically, we tested four sets of hypotheses, linking fence removal and key demographic and environmental predictors to elephant spatial, behavioural, physiological and vegetation responses. We predicted that (1) fence removal would alter elephant spatial dynamics, including increased movement flexibility, space use across the landscape and intergroup proximity; (2) fence removal would alter elephant behavioural dynamics, including reduced aggression and disturbance‐related behaviour, alongside increased affiliation and altered activity budgets; (3) fence removal would alter physiological dynamics by reducing stress‐related biomarkers (fGCM); and (4) fence removal would alter vegetation dynamics due to reduced browsing pressure.

## 2 Methods

### 2.1 Study Area

**FIGURE 1:**
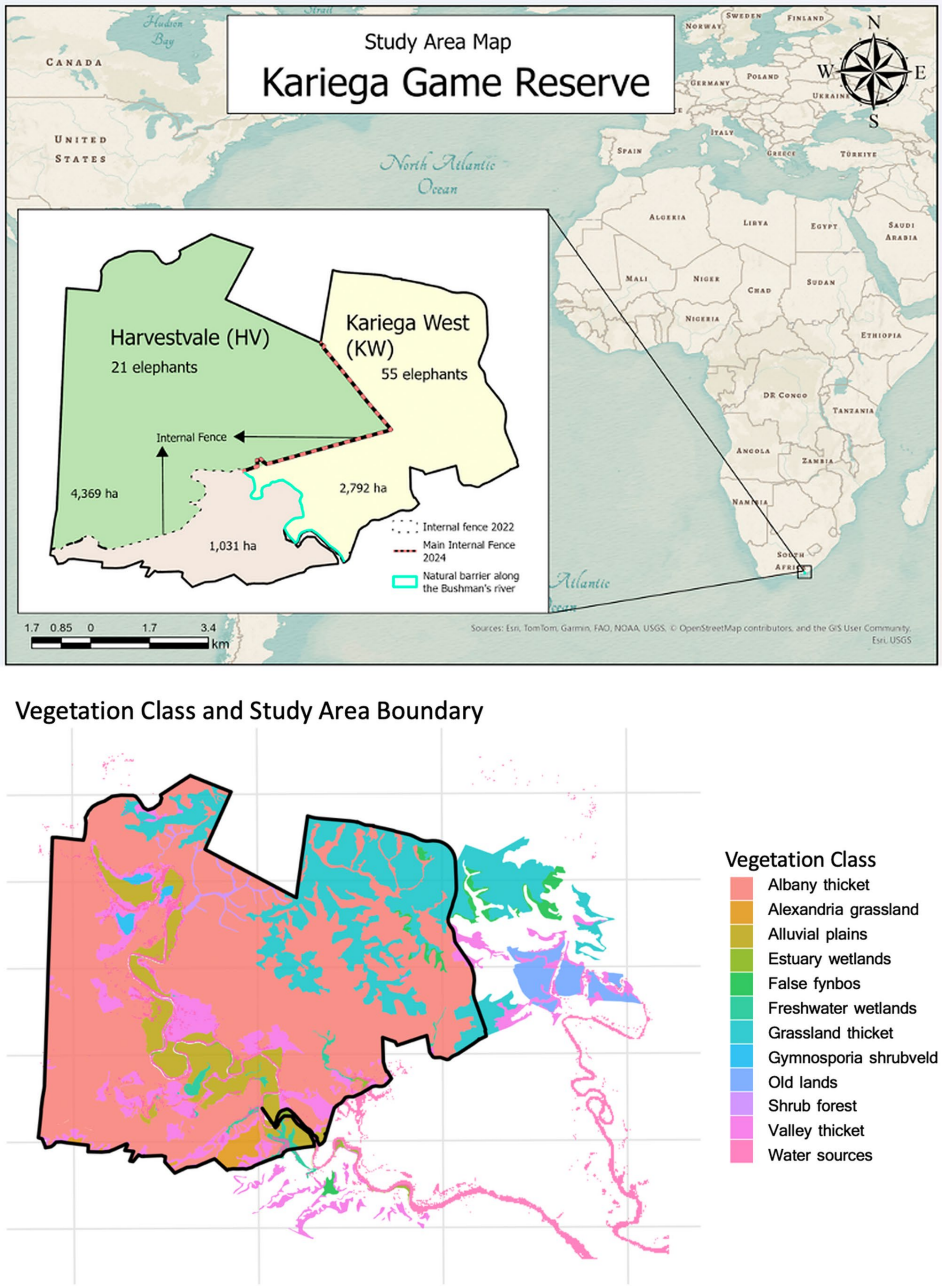
(TOP): Study area map of Kariega Game Reserve, Eastern Cape, South Africa. The reserve is divided into two former management sections, Kariega West and Harvestvale, separated by an internal fence. The inset map shows the location of Kariega Game Reserve within South Africa. The map shows Harvestvale (HV; 4369 ha, 21 elephants; ~4.8 elephants/km^2^) and Kariega West (KW; 2792 ha, 55 elephants; ~19.7 elephants/km^2^). The black dashed line indicates the location of the internal fence prior to removal in Sept 2022 (which added 1031 ha to HV but did not permit herd integration from KW), and the red dashed line shows the fence removed in Dec 2023/Jan 2024 allowing the previously separated populations of KW and HV to access a contiguous shared 8192 ha area for the first time. For the collared elephants in this study: [H1 = Herd 1; Bukela from KW; H2 = Herd 2; Half‐Moon from KW; H3 = Herd 3; Beauty from HV]. (BOTTOM): Vegetation classification map of the study area showing the spatial distribution of major habitat types within the reserve boundary (black outline), used to assess habitat availability, heterogeneity and patterns of elephant habitat use.

The research was conducted at Kariega Game Reserve (KGR), Eastern Cape, South Africa (Figure 1), an 11,500 ha (115 km^2^) privately owned reserve within the Albany Thicket Biome, which is characterised by dense, woody, semi‐succulent vegetation with an average canopy height of 2–3 m. The reserve is bordered by the Kariega and Bushman's rivers and is located approximately 9.5 km from the Indian Ocean, resulting in a marine‐mild climate with annual temperatures ranging from 8°C to 31°C. Rainfall is generally evenly distributed throughout the year, averaging 86.3 mm annually over 128 rainy days (Hoare et al. 2006).

At the study's inception, KGR was divided into four fenced sections, with elephant populations physically separated into two regions: Kariega West (KW; 2792 ha) and Harvestvale (HV; 4369 ha). In total, approximately 75 elephants were distributed among five matriarchal herds across the reserve, with three herds occurring in KW and two herds in HV (Figure 1). Of these, three matriarchal herds were the focus of GPS collar‐based analyses in this study: two herds, Herd 1 and Herd 2 in KW (H1: Bukela; Herd 2: H2: Half‐Moon) and one herd, Herd 3 in HV (H3: Beauty), while the remaining herds were uncollared but included in physiological and behavioural assessments. Available translocation records indicate that these matriarchal herds originated from three distinct source populations and were introduced as intact family units rather than artificially assembled orphan groups. Specifically, the Beauty herd (H3) was translocated from Shamwari Game Reserve in 2014 and occupied HV prior to fence removal, while the Bukela (H1) and Half‐Moon (H2) herds were translocated from Sabi Sands Game Reserve in 2004 and occupied KW. There is no evidence that matriarchal herds occupying KW and HV had prior social association before their translocation to KGR, and they remained spatially segregated by internal fencing prior to fence removal.

KGR removed two internal fences in 2022 and 2023/2024 to better achieve its conservation objectives (Figure 1). In September 2022, 1031 ha were added to HV following the first fence removal, increasing the size of HV to 5400 ha. In January 2024, the primary internal fence separating KW and HV was removed, creating a contiguous 8192 ha area accessible to the entire population for the first time (Figure 1). Prior to fence removal, elephant densities differed markedly between sections (KW: higher density; HV: lower density). Free‐ranging elephants in similar habitats typically occupy annual home ranges of approximately 15,000–30,000 ha (150–300 km^2^; Thouless 1996; Thomas et al. 2008), well exceeding the size of fenced reserve sections at KGR, making internal fence removal a substantial increase in available space for the elephant population but did not increase the total size of KGR.

### 2.2 Data Collection

#### 2.2.1 Movement and Intergroup Proximity

To monitor spatial responses to fence removals, six African savanna elephants were initially equipped with XL LoRa GPS collars (African Wildlife Tracking) in August 2022, approximately 3 months before the first internal fence removal and 16 months before the final integrative fence removal in January 2024. Collared individuals included three adult matriarchs representing known family groups: two from Kariega West (H1 Bukela and H2 Half Moon) and one from Harvestvale (H3 Beauty), and three adult bulls (two from Kariega West and one from Harvestvale; Figure 1). Collars recorded GPS locations at 30 min intervals, enabling detailed assessment of daily movements. Location data were monitored remotely via the LoRa system using EarthRanger software (Vulcan Inc 2025).

An unexpected issue of collar twisting in all three collared bulls within the first 6 months of collaring occurred, necessitating their removal and therefore data on collared bulls was omitted from the study. Twisting incidents were extensively assessed and attributed to browsing behaviour in dense vegetation by males and flaws in collar design (Friswold et al. 2023). Additionally, 4 months after the second fence removal, the matriarch from HV (H3) began having intermittent lapses in GPS data, although still transmitting semi‐regularly.

#### 2.2.2 Behavioural Focal Sampling

Fourteen minute focal samples were recorded via the ZooMonitor application (Lincoln Park Zoo 2019) which allows for simultaneous sampling of continuous (duration‐based) and all‐occurrence (count‐based) behaviour using a pre‐developed ethogram adapted from established references (Langbauer Jr 2000; elephantvoices.org, Elephant Voices 2018; Poole and Granli 2021; Szott et al. 2019; Pretorius et al. 2023; Appendix S1). C*ontinuous sampling* (duration‐based) was used to quantify activity budgets and *all‐occurrence sampling* (count‐based) was used to quantify frequencies of short‐duration behaviours (See Appendix S1 for the complete list of behaviours). Behaviours recorded during all‐occurrence and continuous focal sampling were sorted into behaviour categories that included relaxed, affiliative, vigilant, disturbance‐related, passive‐aggressive and active‐aggressive behaviours (Appendix S1). Disturbance‐related and vigilant behaviour were categorised as responses reflecting heightened awareness, agitation, or avoidance of perceived stimuli rather than direct indicators of physiological stress, and therefore do not necessarily indicate harmful or noxious conditions. Alongside behavioural data, observers recorded environmental (GPS location, vegetation type, weather), anthropogenic (number of vehicles, observer distance) and demographic (sex, age class, elephant ID if known, herd size) variables.

Observations were conducted from vehicles by the principal investigator, KGR staff and trained field assistants using standardised focal sampling protocols (Altmann 1974). Focal individuals were pseudo‐randomly selected, and observers maintained a target distance of ≥ 30 m. Only adult (≥ 15 years) and subadult elephants (9–14 years) were included in behavioural sampling to maintain consistency. A maximum of 180 min of observation occurred per herd daily, and individual focal samples were sampled only once per day per elephant. Behavioural sampling occurred across two sampling periods, approximately 16 months before and 15 months after internal fence removal (Table 1). These included weekly focal sessions (5–10 observations) and biannual intensive daily sampling for 20 days/year. A minimum sample size of 67 focal sessions for the population and study period was established with a 95% confidence level via a power analysis (Taborsky 2010). Due to field constraints, sampling effort varied across years making standard deviations of monthly effort large, particularly in 2024 (SD = 34.27), indicating uneven sampling intensity across months within certain years. Despite this, sufficient coverage was obtained to analyse behavioural trends. Because of this, effort distributions were accounted for in subsequent modelling to ensure robustness of the behavioural analyses.

**TABLE 1:** Summary of focal sampling effort by year, including total focal samples, mean and standard deviation of monthly sampling effort. Note that 2025 was only 6 months of data collection and that years 2022 and 2023 represent pre‐fence removal and years 2024 and 2025 represent post‐fence removal periods.

Year	Total focal samples	Mean monthly samples	SD monthly samples
2022	106	8.83	13.95
2023	213	17.75	23.53
2024	197	16.42	34.27
2025	40	3.33	11.55

A collaborative effort between KGR, Bring The Elephant Home and the Elephant Reintegration Trust developed an elephant identification database to support research and management. Individual IDs enabled longitudinal tracking of movement, behaviour, physiological responses and social roles. Identification followed a modified SEEK protocol (Bedetti et al. 2020) adapted to include herd associations and life‐history details.

#### 2.2.3 Stress‐Related Biomarkers

To assess physiological responses to fence removal, we measured faecal glucocorticoid metabolite (fGCM) concentrations in elephant dung as a non‐invasive indicator of adrenocortical activity (Viljoen et al. 2008; Jachowski et al. 2016). FGCM concentrations represent adrenal activity of the previous ~24–48 h rather than immediate responses to behavioural events (Abraham et al. 2021). Fresh dung samples (< 1 h post‐defecation) were collected from adult (15+ years) and subadult elephants (9–14 years) in both sections of the reserve (HV and KW) before and after main fence removal. Faecal samples were collected opportunistically between June 2021 and December 2024, spanning both wet and dry seasons. Samples collected from 2021 to 2023 were classified as pre‐removal, and those from 2024 as post‐removal. In total, 164 samples were included in the final endocrine dataset (pre: KW = 43, HV = 48; post: 74), exceeding the minimum sample size determined by a power analysis. Samples were collected from both males and females, with efforts to balance sampling across sex and reserve sections.

All samples were stored at −18°C until laboratory analysis. Previously frozen faecal samples were lyophilised, pulverised and sifted using a nylon mesh strainer to remove fibrous material (Fieß et al. 1999). Between 0.050–0.055 g of the faecal powder was then extracted with 80% ethanol in water (3 mL). The suspensions were vortexed for 15 min and subsequently centrifuged at 1500 *g* for 10 min (Ganswindt et al. 2002). The supernatants formed were transferred into microcentrifuge tubes and stored at −20°C until further processing. The resulting extracts were measured for immunoreactive fGCM concentrations using an 11‐oxoetiocholanolone enzyme immunoassay (EIA), detecting fGCMs with a 5*β*‐3*α*‐ol‐11‐one structure. This EIA has been shown to reliably detect adrenocortical function in African elephants (Ganswindt et al. 2003). Detailed assay characteristics, including full descriptions of the assay components and antibody cross‐reactivities, are provided by Möstl and colleagues (2002). Sensitivity (at 90% binding) of the assay was 1.5 ng/g dry weight (DW). Intra‐assay coefficients of variation (CV) determined by repeated measurements of high and low value quality controls were 6.70% and 6.89%. Inter‐assay CV determined by repeated measurements of high and low value quality controls were 7.85% and 12.01%. Serial dilutions of faecal extracts gave displacement curves that were parallel to the respective standard curve with a relative variation of the slope of the trend lines < 3%. All steroid concentrations are expressed per mass of faecal DW matter. All analyses were conducted at the Endocrine Research Laboratory, University of Pretoria with assay procedures following (Ganswindt et al. 2002).

### 2.3 Data Analyses

A combination of spatial processing, remote sensing and statistical analyses was used to evaluate movement and intergroup proximity, vegetation dynamics, behavioural responses and physiological indicators.

#### 2.3.1 Movement and Intergroup Proximity

Spatial and Landsat analyses were conducted using ArcGIS (Esri 2023) following the uploading of GPS location data from EarthRanger to quantify spatio‐temporal changes associated with the increased habitat availability. The dataset included three herds: Herd 1 (H1; Bukela, from KW), Herd 2 (H2; Half‐Moon; from KW) and Herd 3 (H3; Beauty, from HV); note that the initial fence removal expanded range availability only for H3 and did not allow herd integration. Movement behaviour was compared across herds from KW (greater elephant and tourist density) and Harvestvale, which is less densely populated (HV; Figure 1). A GIS database was created, containing the telemetry data, as well as the relevant attributes of Kariega Game Reserve, including fences and reserve section boundaries. Using ArcGIS Pro version 3.5.2 (Esri 2023), each dataset was projected into Hartebeesthoek94 Lo27, the datum currently used for South Africa (Department of Rural Development and Land Reform, n.d.). Additional attributes were then added to the telemetry dataset. Each data point was categorised as pre‐ (4 August 2022 to 9 December 2023), interim‐ (10 December 2023 to 8 February 2024) and post‐fence removal (9 February 2024 to 14 July 2025). Seasonal attributes were also assigned: Spring (1 September to 30 November), Summer (1 December to 28 February), Autumn (1 March to 31 May) and Winter (1 June to 31 August). Kernel density estimation (KDE) was used to quantify seasonal space use and relative habitat utilisation before and after the main fence removal. KDE is a standard approach for home‐range and utilisation distribution analyses in movement ecology (Worton 1989; Fleming et al. 2015; Péron 2019). For each elephant and season, KDEs were generated in ArcGIS Pro using the Kernel Density tool, with a 5 m cell resolution and a 300 m search radius (Esri 2023).

KDE outputs were used in two complementary ways: (1) to visualise and interpret relative habitat use and spatial redistribution across the landscape, and (2) to derive quantitative home range estimates based on utilisation distribution thresholds (50% and 95%). Core and general home range estimations were then created for the collared elephants from the kernel density surface rasters. Using ArcGIS Pro's Extract Values to Points tool, a density value was extracted from the KDE raster and assigned to each GPS tracking point. Points falling in the 50th percentile value range were considered the Core Home Range, and those falling into the 95th percentile made up the General Home Range (Powell 2000; Vander Wal and Rodgers 2012). The home ranges pre‐ and post‐fence removal were then compared with the ArcGIS Pro Intersect tool to determine the area of overlap.

To assess changes in proximity associations among elephant herds following fence removal, GPS collar‐derived proximity alerts were analysed across pre‐ and post‐fence removal periods (when collared elephants were < 30 m of one another; Wittemyer et al. 2005), indicating potential interaction. Alerts were categorised into pre‐fence removal (baseline) and post‐fence removal phases. To examine longer‐duration associations beyond proximity events, a Co‐traveller Analysis was conducted in ArcGIS Pro (Esri 2025a), which compares time‐stamped location data to identify elephants moving together through shared space. Co‐travellers were defined as individuals remaining within 300 m of one another for ≥ 60 min which approximates typical spatial cohesion of elephant groups during coordinated movement and foraging (Fishlock and Lee 2013) while remaining well above GPS positional error (< 20 m), thereby minimising false associations. The ≥ 60‐min minimum duration was selected to capture sustained coordinated movement or shared space use consistent with definitions used in other large‐herbivore movement studies (Wall et al. 2014). Elephants were no longer considered co‐travellers if they were separated by more than 300 m for over 30 min, which also aligned with the 30 min interval collection period of the GPS collars and supporting literature (Archie et al. 2006). Each co‐travelling event was grouped into pre‐ and post‐fence removal periods, and the frequency of these pairwise associations was used as a proxy to evaluate whether time spent together between individual elephants changed following fence removal.

#### 2.3.2 Vegetation Imaging

We calculated a normalised difference vegetation index (NVDI) for the same time period in each of the 3 years (2022–2024) of the study to compare vegetation changes between areas of elephant presence and absence (control) after fence removal, which increased elephant access across the reserve. NDVI is a proven method for calculating biomass and vegetation greenness using satellite data (Tucker 1979; Gandhi et al. 2015). Previous research shows that African savanna elephants preferentially use areas with higher NDVI values (Duffy and Pettorelli 2012), reflecting their selection for greener, more productive vegetation and not necessarily elephants increasing NDVI values. Landsat 9 satellite imagery was obtained at a 30‐m resolution for 3 September 2022, 29 August 2023 and 31 August 2024. The 3 September 2022 image was selected for the baseline image as it was the closest in date to the final elephant collaring with the least amount of cloud cover (< 10%). The 2023 and 2024 images were then chosen with the same date and cloud cover parameters. Using ArcGIS Pro, bands one through six were combined into a composite image for each year, then resymbolised into a natural colour composite. These composite rasters were then clipped to the KGR boundary. Next, the NDVI raster function in ArcGIS Pro was run to calculate the vegetation biomass and greenness for each year's composite image.

The scientific output option was chosen to normalise the value ranges on a −1.0 to +1.0 scale. A high NDVI (> 0.6) indicates dense vegetation; a mid‐range NDVI (0.2–0.6) indicates moderate vegetation density, and a low NDVI (0.0–0.2) represents sparse vegetation or other surfaces, such as water (Carvalho and Campbell 2023a, 2023b; Esri 2025b). To determine changes in vegetation greenness and mass from 1 year to the next, the difference in NDVI values was calculated for 2022 to 2023, 2023 to 2024 and for 2022 to 2024. ArcGIS Pro's raster calculator was used to subtract the values of the earlier year from the values of the later year. The results were then reclassified to reflect a gain, loss, or no change in vegetation. Values that were < = −0.001 were classified as a loss; values between −0.001 and 0.001 represent no substantial change in vegetation; and values > 0.001 were classified as a gain in vegetation (Carvalho and Campbell 2023a; Esri 2025b). Because NDVI reflects vegetation greenness and density rather than forage quality or plant species composition, it should be interpreted as a landscape‐scale proxy for vegetation dynamics rather than a direct measure of elephant browsing impacts or preference.

#### 2.3.3 Behavioural Focal Sampling

We assessed the behavioural responses of elephants to fence removal at KGR between January 2022 and June 2025 (Table 1) using a combination of focal behavioural sampling, activity budget analyses, ordination methods and generalised linear modelling. Behavioural data for elephants in the reserve (collared and uncollared) were collected via 14 min focal samples across internal reserve sections that were historically separated by an internal fence and included both all‐occurrence (count‐based) and continuous (duration‐based) sampling. Continuous (duration‐based) sampling was used to derive activity budgets for the sampling period (Figure 3), while all‐occurrence (count‐based) sampling was used to quantify behavioural response frequencies (Table 2). To evaluate independence among demographic, environmental and contextual variables, we performed Spearman correlation analyses. No pairwise correlation exceeded |r| = 0.5, indicating that multicollinearity was not a concern for subsequent multivariate modelling.

**TABLE 2:** Results of generalised linear mixed models (GLMM) assessing the influence of fence removal and other predictor variables on the rates of: Active aggressive, passive aggressive, disturbance‐related, vigilance and affiliative behaviours. Outcomes depict the overall population and include the variables that proved most influential in the GLMM. β̂ = estimated regression coefficient, *p* = statistical significance and ΔAIC represents the change in Akaike Information Criterion relative to the null model. Bolded values indicate statistically significant effects (*p* < 0.05). GLMMs include both pre‐ and post‐fence removal data, with pre‐removal observations serving as the baseline for comparison. Percentage changes 300 days after fence removal are provided to illustrate behavioural shifts over time. See Appendix S1 for behavioural categories description. The variable ‘Fence removed’ represents a binary comparison between pre‐ and post‐removal periods, with pre‐removal data serving as the baseline. The variable ‘Days since fence removal’ represents a continuous temporal covariate describing gradual change in behaviour as time progressed following fence removal and is distinct from the ‘Change 300 days after fence removal’ row, which summarises model‐predicted proportional change at 300 days post‐removal.

Variable	Active aggressive			Passive aggressive			Disturbance‐related			Vigilance			Affiliative		
	*β*^	*p*	ΔAIC	*β*^	*p*	ΔAIC	*β*^	*p*	ΔAIC	*β*^	*p*	ΔAIC	β^	*p*	ΔAIC
Fence removed	1.872	**< 0.0001**	21.2	−0.23	0.32	−1	0.478	**< 0.0001**	61.8	0.215	**0.017**	3.5	1.229	**< 0.0001**	38.7
Days since fence removal	−0.007	**0.001**	10.7	0	0.742	−1.9	−0.001	**< 0.0001**	15	0.003	**< 0.0001**	49.8	−0.002	**0.02**	3.4
Sex = Cow	−1.078	**0.036**	3.3	0.101	0.489	−1.5	−0.074	**0.091**	0.9	−0.353	**< 0.0001**	31.4	−0.792	**< 0.0001**	24.3
Non‐adult	1.944	**< 0.0001**	30.4	0.417	**0.004**	7.5	−0.101	**0.025**	21.5	−0.209	**0.001**	15.8	0.165	0.246	0.3
# of elephants	0.033	**0.03**	2	0.021	**0.001**	7.1	0.009	**< 0.0001**	14.5	0.002	0.624	−1.8	0.044	**< 0.0001**	51.1
Distance to observer	−0.023	**0.057**	1.8	−0.015	**0.003**	7	−0.007	**< 0.0001**	20.2	−0.007	0	12.1	0.004	0.368	−1.2
Change 300 days after fence removal	−20%			−35%			12%			103%			66%		

To quantify behavioural responses to fence removal, we fitted Poisson Generalised Linear Mixed Models (GLMMs) with log link functions. Response variables included the frequency of active aggressive, passive aggressive, disturbance‐related, vigilance and affiliative behaviours. Fixed effects included fence status (pre/post removal), sex, days since fence removal, age class (adult vs. non‐adult), group size (number of elephants visible) and distance to humans (m). Predictor variables were selected a priori based on established drivers of elephant behaviour and stress. Elephant identity was included as a random intercept to account for repeated observations of individuals, and an offset term for observation duration was used to correct for variation in sampling effort. Models were fitted with pooled (with sex included as a predictor) and separated by sex due to sex differences in social structure and behaviour. Herd‐level random effects were not included because herd membership was not consistently identifiable across sampling periods. To validate findings, GLMs with identical fixed‐effect structures and offset terms were fitted as supplementary analyses which corroborated the direction and magnitude of behavioural responses identified in mixed‐effects models. To estimate behavioural adjustment over time, we calculated predicted behavioural change 300 days after fence removal using:

Δy^300=βFence removed+300×βDays after removal

The temporal predictor was capped at 300 days to reflect an asymptotic adjustment assumption and avoid unrealistic linear extrapolation while providing a standardised estimate of long‐term behavioural response.

Activity budgets were computed for the full population and separately for bulls and cows and pooled for subadults and adults as no significant difference was observed for age class. To quantify uncertainty around mean activity proportions, we generated 95% confidence intervals through non‐parametric bootstrapping (100,000 iterations). Pre‐ and post‐fence removal changes in foraging, locomotion, resting, self‐maintenance and vigilance durations were evaluated using paired sample *t*‐tests and linear regression models. All analyses were conducted in R (R Core Team 2022) using the packages lme4, car, ggplot2, vegan and boot.

#### 2.3.4 Stress‐Related Biomarkers

fGCM (μg/g dry weight) was used as the response variable to evaluate whether alterations in stress‐associated hormone concentrations occurred between samples collected pre and post fence removal. The primary predictor of interest was fence status (pre‐ and post‐removal) with additional explanatory variables including sex, age class (adult [A; ≥ 25 years], young adult [YA; 19–25 years] and subadult [SA; 9–15 years]), seasonal precipitation (wet: October–March; dry: April–September), meteorological season (summer: December–February; autumn: March–May; winter: June–August; spring: September–November), temperature (°C) and rainfall (mm). Initial sample‐level comparisons between fence status periods were conducted using Welch's *t*‐tests and Mann–Whitney U tests. To estimate the association while accounting for potential covariates, we fitted ordinary least squares (OLS) linear regression models. A series of nested models was explored, including an unadjusted model with fence status alone and progressively adjusted models with the above variables. Model fit among candidate models was evaluated using Akaike's Information Criterion (AIC), adjusted R^2^ and Bayesian Information Criterion (BIC). Daily rainfall and temperature data using the GPS coordinates of KGR were extracted from the NASA POWER climate archive (NASA POWER Project 2024) and cross‐checked with ERA5‐Land data (Muñoz Sabater 2019) summarised as mean temperature and rainfall. We also evaluated whether adult outliers might influence model inference using a standard interquartile‐range criterion, with one adult outlier identified and excluded.

Because some individuals were sampled more than once, potential non‐independence among samples was evaluated. A mixed‐effects model including elephant identity as a random intercept was initially fitted to account for repeated observations, but sparse and unevenly distributed samples resulted in a singular model fit. To assess potential pseudoreplication, a conservative sensitivity analysis averaged repeated samples within individual and period so that each elephant contributed at most one value per period. The resulting sensitivity analysis showed the same directional pattern, with lower fGCM concentrations after fence removal, indicating that the primary inference was robust to potential pseudoreplication. Analyses were conducted in Python using pandas, scipy, statsmodels and seaborn (Python Software Foundation 2024).

### 2.4 Permits

Research permission was granted by KGR, and all elephant collaring activities were undertaken as a reserve‐led wildlife management action under KGR's standing Threatened or Protected Species (TOPS) permits (TS‐202308000003999) in compliance with National Environmental Management Biodiversity Act 2004. The immobilisations were not initiated or performed by researchers, but were conducted solely under the reserve's legal management authority. A formal letter from KGR confirming the management nature of the immobilisations, including veterinary oversight, is on file. Non‐invasive components of this study adhered to all applicable ethical guidelines. Behavioural observations involved no manipulation or disturbance to elephants and were conducted in compliance with KGR protocols and conducted under KGR staff and therefore did not require institutional animal care approval. Dung sample collection was approved under The University of Pretoria, Faculty of Veterinary Science, Animal Ethics Committee (REC254‐19) which is registered with the National Health Research Ethics Council (NHREC) of South Africa.

## 3 Results

### 3.1 Movement and Intergroup Proximity

**FIGURE 2:**
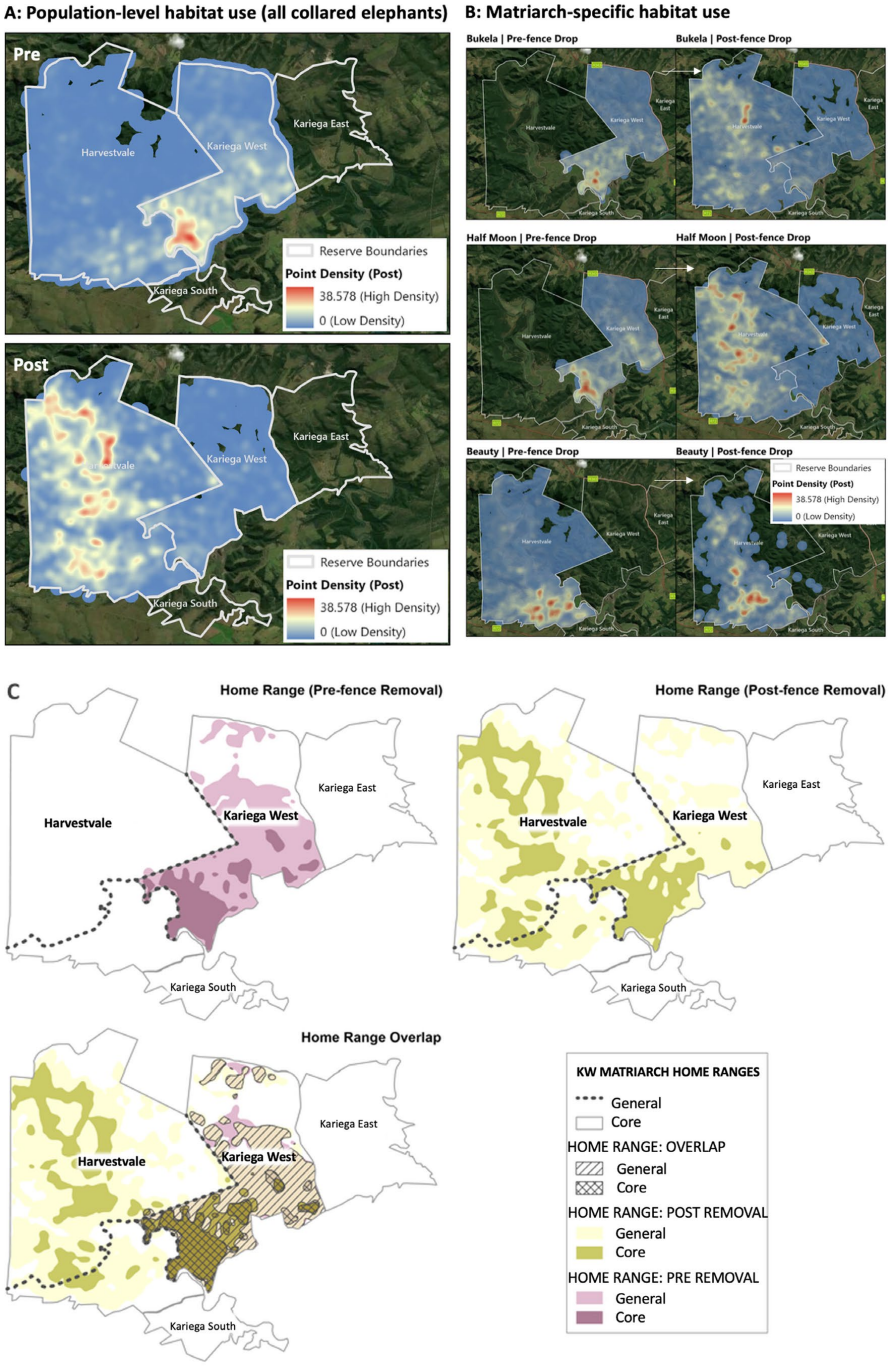
(A) (LEFT): Habitat usage by all collared elephants pre‐fence removal (top) and post fence removal (bottom) at Kariega Game Reserve. (B) (RIGHT): Elephant spatial distribution pre (left) and post (right) fence removal across Kariega Game Reserve for the three collared matriarchs individually Bukela from Kariega West (top); Half Moon from Kariega West (middle); Beauty from Harvestvale (bottom). Sources: Esri, TomTom, Garmin, FAO, NOAA, USGS, OpenStreetMap contributions and the GIS user Community. (C) Matriarchal elephant home ranges before and after internal fence removal at Kariega Game Reserve. Upper panels show general (95% kernel) and core (50% kernel) home ranges prior to fence removal (left) and following fence removal (right). The lower panel illustrates spatial overlap between pre‐ and post‐removal home ranges, highlighting areas of retained use and newly incorporated habitat. Dashed lines indicate internal fences removed during the study period.

H1 (KW) took 64 days to cross and 92 days to fully occupy the expanded section. In contrast, H3 (HV) did not cross into the newly available habitat until ~15 months after fence removal. Overall, movement patterns indicated a general shift from the more densely populated sector of KW towards the lower‐density area of HV, with only limited movements in the opposite direction (Figure 2A,B).

Following internal fence removal, elephants exhibited substantial expansion of both general (95%) and core (50%) home ranges for the two matriarchal herds from KW (H1 and H2; Figure 1; Figure 2C). Kernel density estimates showed an approximate 30% median increase across collared elephants in annual utilisation distribution area following fence removal. Individual‐level responses were pronounced: H1's general home range more than doubled from 1279.9 ha to 3291.1 ha (+2011.2 ha), and core range from 460.1 ha to 1095.0 ha (+634.9 ha); H2's general home range from 1290.9 ha to 2902.6 ha (+1611.7 ha), and core range from 471.9 ha to 1057.6 ha (+585.7 ha). The averaged total general home range area increased from 3264.0 ha to 3432.3 ha post‐removal (+168.3 ha), while core home range area expanded more markedly from 898.4 ha to 1217.3 ha (+318.9 ha). Despite high increase in elephants from KW, the average increase was reduced due to H3, who originally occupied the less densely populated side of the reserve (HV) and increased her range less drastically. Spatial overlap analyses indicated limited reuse of pre‐removal space. The combined matriarchs shared 1034.3 ha of general home range overlap and 434.7 ha of core overlap across periods, while overlap between pre‐ and post‐removal general (166.7 ha), and core ranges remained low (80 ha). Kernel density mapping corroborated these quantitative results, showing clear post‐removal expansion into Harvestvale and across former internal fence alignments, alongside increased continuity between previously segregated reserve sections (Figure 2C).

Prior to fence removal, elephant habitat use was largely concentrated in the southern section of the reserve within both HV and KW (Figure 2A). Following fence removal, elephants expanded the spatial extent of habitat use and redistributed their movements across a larger proportion of the landscape with reduced localisation of hotspot areas (Figure 2A–C). The yearly average extent of habitat use shifted northward, westward, centrally and into newly accessible habitat (Figure 2A–C). When evaluated at the herd level, visual inspection of the kernel density within the maps reveals distinct spatial shifts in elephant distribution after fence removal for KW elephants and reduced localised hotspots (Figure 2B). Post fence removal all three collared elephants expanded their movements beyond former boundaries and exhibited increased overlap between previously isolated zones, particularly for those formerly inhabiting the more densely populated region of the reserve (H1 and H2 from KW).

Seasonal movement and occupation patterns became broader and more evenly distributed across the landscape following fence removal, with all herds expanding their seasonal ranges and increasing inter‐seasonal connectivity (Figure S1). Prior to removal, herds showed strong seasonal fidelity, particularly in the southern section in winter. After removal, spring and summer ranges expanded northward and westward, while autumn and winter movements extended into central, northern and eastern areas. H2 (KW) exhibited the greatest spatial expansion, forming a continuous movement network across much of the reserve, while H1 (KW) and H3 (HV) also expanded beyond their former southern or southwestern ranges, particularly during autumn, winter and spring. Across all herds, post‐fence removal seasonal movements encompassed a more heterogeneous set of habitats while maintaining limited use of previously saturated southern areas.

Patterns of elephant co‐travelling (> 300 m proximity for ≥ 60 min) varied among matriarchal groups and differed markedly post‐fence removal. Before fence removal, proximity events occurred almost exclusively between the H1 and H2 herds, which occupied the same side of the barrier, with only occasional detections when herds used adjacent areas across the fence. Following fence removal, proximity events increased substantially across all matriarchal pairs. The H1–H2 pairing, which had not been previously separated, showed the greatest increase, while the previously separated H2–H3 pair exhibited a strong rise in association frequency and H1–H3 (previously separated) increased modestly. During the immediate post‐removal transition period, proximity events remained low, indicating a potential lag in herd redistribution, but subsequently stabilised at higher levels ~6 months post removal.

### 3.2 Behavioural Responses

A comprehensive population register and identification database was developed for the elephant population within KGR, resulting in complete identification profiles for 69 of the ~76 known individuals (some elephants > 5 > years lacked necessary identifying features).

#### 3.2.1 All‐Occurrence

A total of 607 14‐min focal samples of behaviour were collected between January 2022 and June 2025 (Table 1). A collinearity assessment indicated that most behavioural and contextual variables were sufficiently independent for statistical analyses, with no pairwise correlations exceeding |r| = 0.5, confirming that multicollinearity is not a major concern for subsequent multivariate modelling.

Overall, active aggressive behaviour increased immediately after fence removal (*β* = 1.87, *p* < 0.0001) but then declined by 20% over the 300‐day post‐removal period (Table 3). Non‐adults exhibited increased aggression (*β* = 1.94, *p* < 0.0001), as well as larger elephant groups (*β* = 0.033, *p* = 0.03; Table 2). By contrast, cows were less aggressive compared to bulls (*β* = −1.08, *p* = 0.036). Passive aggressive behaviour increased significantly in sub‐adults (*β* = 0.42, *p* = 0.004) and with the number of elephants (*β* = 0.021, *p* = 0.001). However, passive aggression decreased by 35% over 300 days post‐fence removal. Disturbance‐related behaviour rose significantly immediately post‐removal (*β* = 0.48, *p* < 0.0001), with increases also associated with time since removal (*β* = 0.008, *p* < 0.0001), larger elephant groups (*β* = 0.009, *p* = 0.001). and sub‐adults (*β* = −0.10, *p* = 0.025). Despite this, disturbance‐related behaviours increased only modestly (+12%) across the 300‐day post‐removal period. Vigilance showed a significant increase following fence removal (*β* = 0.22, *p* = 0.017), rising by 103% within 300 days and was greater in bulls (*β* = −0.35, *p* < 0.0001) and adults (*β* = −0.21, *p* = 0.001). Affiliative behaviour rose significantly post‐fence removal (*β* = 1.23, *p* < 0.0001), with a 66% increase 300‐days post‐removal period. The number of elephants was positively correlated with affiliation (*β* = 0.044, *p* < 0.0001), while cows exhibited fewer affiliative behaviours compared to bulls (*β* = −0.79, *p* < 0.0001).

**TABLE 3:** Parameter estimates from an ordinary least squares (OLS) regression model evaluating predictors of faecal glucocorticoid metabolite (fGCM) concentration (pre fence removal = 91; post fence removal = 73 samples). Explanatory variables included fence status (pre vs. post removal), seasonal precipitation (wet: October–March; dry: April–September), sex, meteorological season (summer: December–February; autumn: March–May; winter: June–August; spring: September–November), age class (adult [A; ≥ 25 years], young adult [YA; 19–25 years], and subadult [SA; 9–15 years]), temperature (°C) and rainfall (mm). Temperature and rainfall were obtained from the NASA POWER climate archive (NASA POWER Project 2024) for GPS coordinates of Kariega Game Reserve, South Africa. Coefficients are presented with 95% confidence intervals, standard errors, *t*‐statistics and *p*‐values. Significant values (*p* > 0.05) are bolded.

Variable	Estimate	Lower CI	Upper CI	SE	*t*	*p*
Fence removal (pre vs. post)	0.0659	0.0172	0.1146	0.0247	2.6731	**0.0083**
Seasonal precipitation (wet vs. dry)	0.1335	0.0455	0.2215	0.0445	2.9973	**0.0032**
Sex (m vs. f)	−0.0802	−0.1217	−0.0386	0.021	−3.8133	**0.0002**
Met. season (spring vs. autumn)	−0.0182	−0.1023	0.066	0.0426	−0.4268	0.6701
Met. season (summer vs. autumn)	0.0222	−0.0368	0.0812	0.0299	0.7433	0.4584
Met. season (winter vs. autumn)	0.0493	−0.0459	0.1445	0.0482	1.0238	0.3076
Age class (subadult vs. adult)	−0.0054	−0.0521	0.0414	0.0237	−0.2263	0.8213
Age class (young adult vs. adult)	0.0104	−0.0415	0.0624	0.0263	0.3972	0.6918
Temperature (per °C increase)	−0.0019	−0.0124	0.0086	0.0053	−0.3504	0.7265
Rainfall (per 1 mm increase)	−0.0045	−0.0093	0.0004	0.0025	−1.8195	0.0708

Additionally, ΔAIC comparisons showed that fence removal and time since removal were the strongest predictors of behavioural change (Table 2). In the combined model, fence removal substantially improved model fit for disturbance‐related (ΔAIC = 61.8), affiliative (ΔAIC = 38.7) and active aggressive behaviours (ΔAIC = 21.2), while days after removal strongly influenced vigilance (ΔAIC = 49.8) and disturbance‐related behaviours (ΔAIC = 15). Bulls exhibited the clearest behavioural response to fence removal, with high ΔAIC values for disturbance‐related (108.4) and affiliation (44.8), and strong temporal effects on vigilance and disturbance‐related behaviours (ΔAIC = 19.9–25.9). Age class was also important for bull aggression (ΔAIC = 23), and group size strongly predicted affiliative behaviour (ΔAIC = 30.7). In cows, days after removal was the dominant predictor of vigilance (ΔAIC = 33.7), and age class strongly affected vigilance and disturbance‐related (ΔAIC = 14.6–17.8), while group size and distance to humans were most influential for affiliative behaviour.

#### 3.2.2 Continuous

**FIGURE 3:**
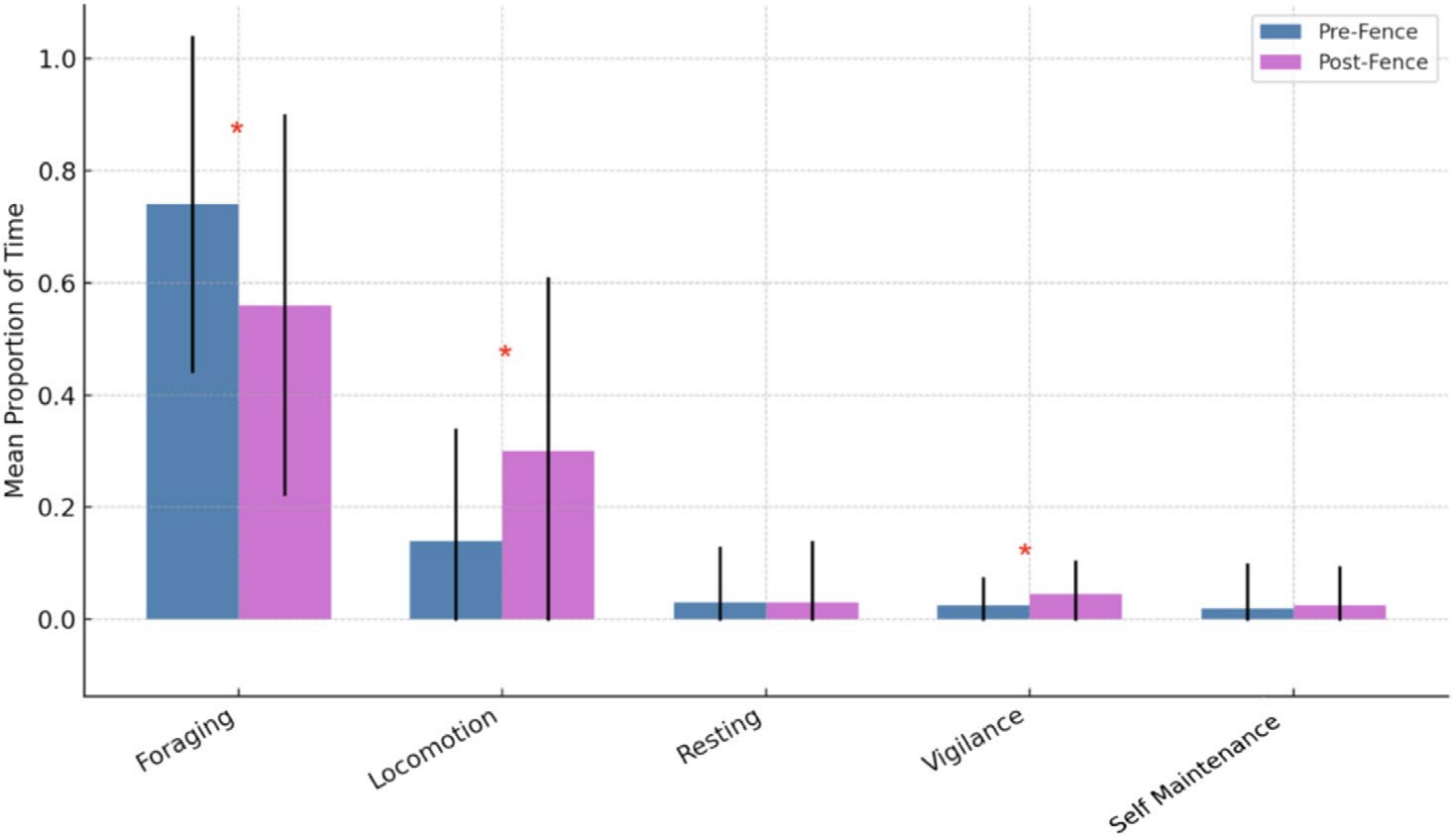
Mean proportion of time spent in continuous (duration‐based) behaviours during 14‐min focal observations representing within‐focal activity budgets rather than full daily activity budgets pre‐ and post‐fence removal with error bars at +/− 1 SD. Foraging includes feeding and drinking, Resting refers to sleeping or lying down, and Self Maintenance refers to dusting, bathing, scratching and wallowing (see Appendix S1). Asterisk indicates statistical significance (*p* < 0.05).

Following fence removal, substantial shifts were observed for continuous behaviours (activity budgets; Figure 3). Foraging (feeding and drinking) decreased by 24.3%, dropping from 74.0% to 56.0% of observed time (*p* < 0.001), while locomotion increased by 108.7%, from 14.5% to 30.2% of observed time (*p* < 0.001). Vigilance behaviour durations also showed a significant increase of 93.6% (1.7% to 3.3%; *p* = 0.027). Effect size estimates (Cohen's d; Cohen 1960) supported these patterns, with locomotion showing the strongest effect (*d* = 0.63), followed by foraging (*d* = −0.56), both within the medium to large range. When separated by sex, locomotion increased significantly for both cows (*p* < 0.001) and bulls (*p* < 0.001). Foraging behaviours differed significantly by sex pre‐ to post‐fence removal (*p* = 0.011), with bulls exhibiting greater reduction in foraging and increased locomotion post‐fence removal.

### 3.3 Stress‐Related Biomarkers

**FIGURE 4:**
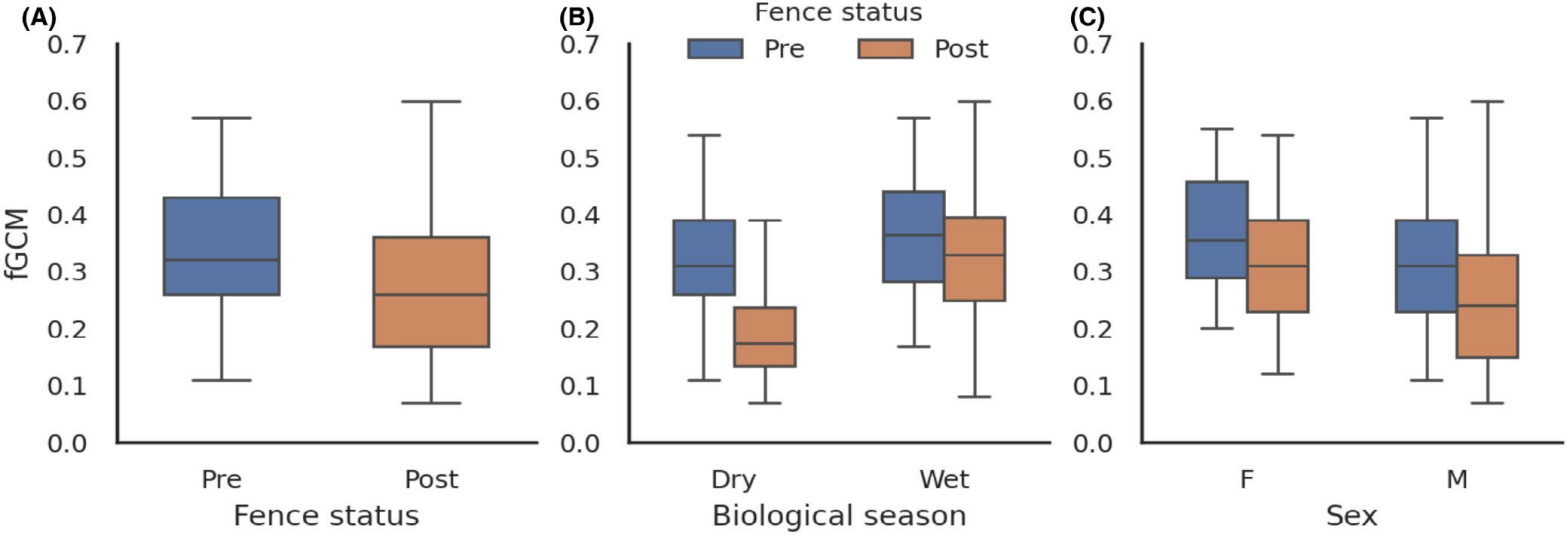
Distribution of faecal glucocorticoid metabolite (fGCM) concentrations of African elephants at Kariega Game Reserve, South Africa (A) fence status (pre to post removal), (B) seasonal precipitation stratified by fence status and (C) sex stratified by fence status. Boxplots show the median, interquartile range and whiskers. fGCM concentrations in overall pre to post, dry season and in males pre to post fence removal achieved statistical significance via an OLS regression (*p* > 0.05). Sample sizes are provided in Table S1.

A total of 164 faecal samples were included in the analysis (pre = 91; post = 73; Table S1). Sample level mean fGCM concentrations were lower post fence removal (pre = 0.349 ± 0.126 SD; post = 285 ± 0.145 SD; *p* = 0.0029). In the OLS regression that included fence status (pre/post), sex, age class (A, YA, SA), seasonal precipitation (wet/dry), meteorological season, temperature and rainfall, fGCM concentrations reduced significantly post fence removal (β = 0.0659, 95% CI 0.0172 to 0.1146; *p* = 0.0083; Table 3; Figure 4). The OLS model using all variables explained 24.7% of the variance in fGCM concentrations (R^2^ = 0.247, adjusted R^2^ = 0.213). This indicates that the decline in fGCM following fence removal remained statistically significant even after controlling for demographic and environmental covariates (Figure 4, Table 3).

Seasonal precipitation was also a significant predictor, with higher fGCM concentrations in the wet season (*β* = 0.1335, 95% CI 0.0455 to 0.2215; *p* = 0.0032; Figure 4; Table 3). However, mean fGCM concentrations in the dry season were significantly higher pre (0.336 ± 0.127, *n* = 53) to post fence removal (0.191 ± 0.080, *n* = 26), with the difference remaining significant after adjusting for sex and age class (*β* = 0.1413, *p* < 0.001). In contrast, the wet season showed a smaller pre to post difference (pre: 0.367 ± 0.125; post: 0.336 ± 0.148; *β* = 0.0392, *p* = 0.187). Sex was also a consistent predictor of fGCM, with females exhibiting significantly higher concentrations than males (*β* = −0.0889, 95% CI −0.1299 to −0.0480, *p* < 0.001); however, fGCM concentrations were significantly higher in males pre to post fence removal (*β* = 0.067, *p* = 0.014), whereas the pre to post fence removal difference was not significant in females (*β* = 0.049, *p* = 0.123; Figure 4; Table 3).

### 3.4 Vegetation Imaging

**FIGURE 5:**
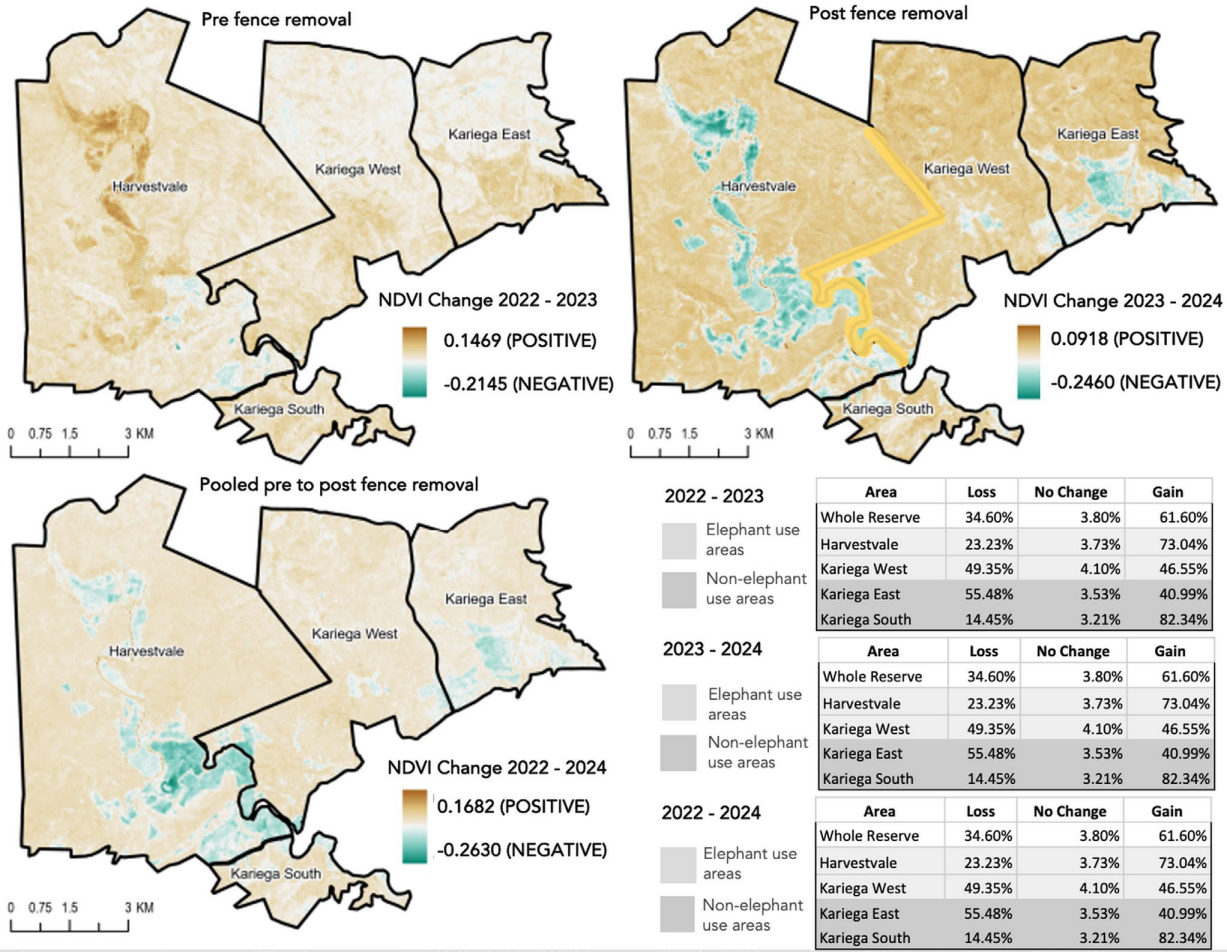
Normalised Difference Vegetation Index (NDVI) year over year change across Kariega Game Reserve (KGR) from January 2022 to December 2024. NDVI was calculated across full annual periods (January–December) for each year, ensuring that comparisons between years reflect equivalent seasonal coverage. Fence removal occurred in December 2023–January 2024. Maps show NDVI change for the pre‐removal period (2022–2023), post‐removal period (2023–2024) and the pooled pre to post comparison (2022–2024). Positive values (brown) indicating vegetation greening and negative values (green) indicating vegetation loss. Reserve sections are delineated and classified as elephant‐occupied areas (Harvestvale and Kariega West; blue outlines) and non‐elephant control areas (Kariega East and Kariega South; red outlines). Tables summarise the proportion of each section exhibiting NDVI loss, no change, or gain in each period. Blurred line in post removal period shows fence removal line. Note that maps depict the magnitude of NDVI change, whereas summary statistics reflect the proportion of area experiencing gain or loss; widespread low‐magnitude declines may therefore appear less visually dominant.

NDVI analyses revealed spatially heterogeneous but broadly comparable vegetation dynamics across elephant‐occupied (HV and KW) and non‐elephant control sections (Kariega East, Kariega South) before and after fence removal (Figure 5). During the pre‐removal period (2022–2023), NDVI gains dominated across the reserve (Total: 61.6% gain, 34.6% loss, 3.8% no change). Elephant‐occupied sections exhibited substantial greening during this period (HV: 73.0% gain and 23.2% loss; KW: 46.6% gain and 49.4% loss). Non‐elephant control sections displayed greater variability but similar results (Kariega South: 82.3% gain and 14.5% loss; Kariega East: 41.0% gain and 55.5% loss; Figure 5), indicating that pre‐fence removal vegetation density was not uniquely associated with elephant presence (Figure 5).

In the post‐removal year (2023–2024), NDVI loss increased markedly across both elephant use and non‐use areas, reflecting a reserve‐wide shift rather than an elephant‐specific response to the increase in elephants' access to previously restricted areas post fence removal (HV = 21 and KW = 55 elephants to 76 across the reserve). Area‐mean rainfall declined between NDVI comparison windows, from approximately 1306.8 mm during the pre‐January 2022–December 2023 period to 1205.8 mm during January 2023–December 2024 post periods (a reduction of ~101 mm; 7.7%; Open‐Meteo 2025). At the reserve scale, 84.8% of the area exhibited NDVI loss and only 12.5% gain, though the scale of loss proportions was small (Figure 5). Elephant‐occupied areas followed this broader pattern (HV loss = 90.6%; KW loss = 76.2%) alongside non‐elephant control areas (Kariega South loss = 91.53%; Kariega East loss = 76.8%), demonstrating that post‐removal NDVI declines were widespread and not confined to elephant‐use areas. Further, annual NDVI maps revealed pronounced interannual variation in vegetation greenness across the reserve between 2022 and 2024, with widespread increases in 2023 followed by declines and greater spatial heterogeneity in 2024. These shifts also occurred consistently across both elephant‐use and non‐elephant sections showing a reserve‐wide temporal signal (Figure 5).

When NDVI change was pooled across the full 2022–2024 period, the majority of the reserve was still classified as NDVI loss (82.6%), with smaller proportions showing gain (13.8%) or no change (3.6%). Again, long‐term vegetation outcomes were broadly comparable between elephant‐use and non‐elephant control sections (elephant‐use areas: mean NDVI loss = 81.3% and gain = 14.9%; non‐elephant areas: loss = 84.5% and gain = 12.9%). At the section level, elephant‐use areas (HV = 15.7% gain; KW = 14.0% gain) exhibited higher pooled gains than non‐elephant controls (Kariega East = 3.9% gain and highest loss of 95.0%; Kariega South = 21.8% gain and 73.94% loss).

## 4 Discussion

This study provides one of the first integrated assessments of how internal fence removal within a subdivided reserve influences elephant movement, inter‐group association patterns, behavioural responses and stress‐related biomarkers, alongside vegetation assessments. By comparing these diverse patterns before and after fence removal, we highlight how restoring landscape connectivity can transform elephant socioecology while improving well‐being and maintaining ecosystem outcomes congruent with regional patterns. Expanded movement can redistribute foraging pressure and enhance elephants' roles as seed dispersers, ecological engineers and nutrient transporters (Reed et al. 2025). With reduced spatial constraints, these ecosystem services are more evenly distributed, promoting spatial heterogeneity, soil fertility and woody vegetation regeneration (Campos‐Arceiz and Blake 2011; Poulsen et al. 2018). Although elephants can have negative impacts on vegetation under high densities or sustained local pressure (Owen‐Smith et al. 2019), restoring connectivity reduces the risk of chronic over‐browsing by dispersing the pressure more evenly across the landscape (Asner et al. 2016). Our findings underscore the conservation value of reconnecting fragmented habitats and demonstrate that even modest increases in connectivity and functional space can yield meaningful benefits.

Spatial findings are broadly consistent with our hypotheses that restoring connectivity would expand movement, reduce spatial constraint and improve both elephant well‐being and ecological function. Fence removal consistently broadened elephant space use, indicating that physical barriers had previously constrained access to portions of the landscape. Once internal divisions were removed, elephants expanded into previously inaccessible areas, redistributed their habitat use and greatly reduced the intensity of localised hotspots and browsing pressure. These changes align with long‐standing evidence that elephants readily respond to new spatial opportunities and that connectivity drives more heterogeneous movement patterns (Graham et al. 2009; Druce et al. 2008; Pandraud et al. 2020). Variation in how quickly herds incorporated new habitat likely reflects group‐level differences in exploratory behaviour shaped by matriarchal decision‐making, local conditions and perceived risk (McComb et al. 2001, 2011; Roever et al. 2013; Pandraud et al. 2020; Bates et al. 2025).

Elephants shifted from areas of higher to lower elephant density and tourism pressure, highlighting density‐dependent movement (Sibly et al. 2003; Young and van Aarde 2010; Szott et al. 2019). This movement was evident in matriarchs from the more densely populated side of the reserve greatly increasing range and engaging in increased exploration which was less evident from the matriarch already occupying the less densely populated region. As predicted, movements became more evenly distributed across the reserve over time, indicating that improved connectivity enabled broader, more continuous ranging. The expansion and reconfiguration of home ranges further demonstrate that internal barriers had previously imposed spatial constraints on both general and core space use (Shannon et al. 2006; Goldenberg et al. 2018). Core ranges expanded disproportionately relative to general ranges, suggesting not only exploratory use of newly accessible areas but their integration into routine movement patterns, supported by low pre to post overlap. Connectivity also reshaped seasonal patterns by weakening strong seasonal fidelity observed pre fence removal (especially in winter) which expanded into newly accessible habitat post removal, reflecting increased seasonal plasticity and more environmentally responsive space use. This aligns with findings that restored connectivity and access to heterogeneous landscapes enhance resource tracking and dynamic movement strategies in wide‐ranging herbivores (Wittemyer et al. 2007; Shrader et al. 2010; Wato et al. 2016; Wambua et al. 2025). These changes support our hypotheses by indicating reduced spatial clustering and localised browsing pressure, more natural space use and enhanced seasonal flexibility, which are key components of ecological resilience in wide‐ranging herbivores (Graham et al. 2009; Asner and Levick 2012).

As predicted, removing internal fences also increased social proximity among groups, demonstrating that the former barrier had limited opportunities for inter‐group interactions. Post‐removal, co‐travelling and proximity events rose substantially across previously separated and non‐separated groups, indicating greater spatial overlap and increased social interaction for all, possibly contributing to well‐being and societal functioning. This is consistent with findings from transboundary landscapes where elephants expand social networks when movement corridors are restored (Naidoo et al. 2022). Despite this increased mixing, core matrilineal bonds remained stable, mirroring long‐term observations that elephant family units are highly cohesive even when broader social opportunities expand (Archie et al. 2006, 2011). Greater social integration may enhance information transfer, genetic mixing, disturbance responses and social cohesion – key factors in small, fenced reserves where isolation can pose long‐term risks (Gobush et al. 2009; Garaï et al. 2023).

As hypothesised, fence removal had a significant and positive effect on elephant behavioural profiles, emerging as the primary driver of behavioural response alongside time since removal. Immediately following removal, elephants exhibited increases in aggression (mostly towards conspecifics), disturbance‐related behaviour, vigilance and affiliation, consistent with an initial exploratory and assessment phase to newly accessible space and social opportunities (Poole and Moss 2008; Chiyo et al. 2011). Over time, active and passive aggression declined lower than pre‐fence removal conditions, suggesting acclimation and reduced overall tension, while disturbance‐related behaviour remained only modestly elevated. In contrast, vigilance and affiliative behaviour remained elevated, suggesting a sustained increase in environmental awareness and social cohesion. Bulls exhibited stronger behavioural responses to fence removal, consistent with greater exploratory and ranging behaviour in males (Poole 1994), but also exhibited reduced aggression over time. Cows also exhibited reduced aggression and disturbance‐related behaviour over time, supporting the interpretation that increased space reduces social tension and promotes more stable group dynamics (Fishlock and Lee 2013). Over time, these behavioural shifts suggest a transition from short‐term disturbance to longer‐term stabilisation, reflecting adaptive responses to expanded space. Elephant group size, age class and proximity to humans further modulated certain behaviours, reinforcing the importance of social and anthropogenic context in shaping responses (Szott et al. 2019; Wall et al. 2021). Activity budgets support movement findings consistent with exploratory behaviour alongside more active and balanced time allocation that shifted from foraging to locomotion, indicating a prioritisation of exploration over resource acquisition (Shannon et al. 2008) resembling elephants in open systems (Makati et al. 2025).

Further supporting our hypothesis, fGCM concentrations declined significantly post‐fence removal indicating reduced stress‐related biomarkers, which remained reduced after accounting for demographic and environmental variation. Females exhibited higher fGCM concentrations than males overall, although males showed a larger reduction post fence removal. This pattern likely reflects sex‐related differences in stress perception and related GC output linked to social structure and reproductive investment (Pokharel et al. 2019), as well as a potential reduced social tension, greater freedom to range in males, or impacts of musth (Poole 1994; Ganswindt et al. 2005a, 2005b). A stronger pre‐ to post‐contrast occurred in the dry season, likely due to reduced resources and higher energetic demands (Foley et al. 2001), though other studies report no seasonal differences (Troup et al. 2022). In fenced systems however, such pressures may be amplified by limited movement across patchy resources (Jachowski et al. 2012). While fGCM concentrations were higher in the wet season, post‐removal reductions did not vary seasonally, possibly reflecting weaker physiological constraints when resources are abundant (Kimuyu et al. 2021; Pokharel et al. 2017). The sustained decline in fGCM following fence removal across sex and age classes suggests benefits of increased space, potentially via reduced crowding, competition and more space to recover from tourism pressure (Szott et al. 2020a), alongside greater movement autonomy. While causality cannot be confirmed, the consistent reduction indicates improved physiological conditions under reduced spatial restriction. This aligns with expanded ranging, more balanced activity budgets and reduced aggression, suggesting well‐being benefits beyond observable behaviour (Jachowski et al. 2013; Szott et al. 2020b).

Also, in support of our hypothesis, vegetation responses following fence removal indicate that redistributed elephant movements did not increase degradation beyond background environmental variability. NDVI patterns were comparable between elephant‐use and non‐elephant control sections before and after removal, suggesting vegetation change was not uniquely associated with elephant presence despite increased access and exploratory use. This suggests that expanded ranging redistributed, rather than intensified, browsing pressure, consistent with ecological theory that connectivity diffuses herbivore impacts across the landscape (Hayward and Kerley 2009; Thompson 2022). Instead, spatially heterogeneous gains and losses aligned with reserve‐wide climatic variation, particularly reduced rainfall in the post‐removal period (Open‐Meteo 2025), reinforcing a climate‐driven, rather than elephant‐driven response in vegetation density, with fence removal enabling movement without exacerbating degradation. However, as analyses were limited to NDVI, finer‐scale changes in plant composition or forage quality may not have been detected.

Several limitations of the current analysis should be considered. Annual variation in sampling effort may have reduced the resolution of fine‐scale behavioural or physiological trends. The post‐removal monitoring period limits inferences about multi‐year stabilisation following increased space. NDVI outcomes were partially confounded by climatic variability, particularly rainfall variation across years. This highlights the need to conduct fence removal in conjunction with vegetation monitoring and seasonal variation, particularly during drought or in sensitive habitats. Variation in collar functioning and sample size may have limited the representation of herd‐level variation due to collar removal in bulls in the study (Friswold et al. 2023) and intermittent GPS points. Because some individuals contributed more than one dung sample, pseudo replication cannot be fully excluded. Although the main pattern was robust in direction to individual‐level aggregation, the reduced statistical support in a sensitivity analysis indicates that the physiological results should be interpreted cautiously. We also acknowledge that fGCM levels may be influenced by factors that were not measured in this study, including habitat quality, diet, reproductive or lactation status, body condition, social dynamics and other environmental stressors (Millspaugh and Washburn 2004; Pokharel et al. 2017; Pokharel et al. 2019; Pokharel and Brown 2023). Future studies could incorporate multi‐year monitoring, camera traps and microhabitat vegetation surveys to capture finer‐scale processes. Longitudinal social network analyses would further clarify how connectivity influences social restructuring and integrating acoustic monitoring could illuminate changes in communication patterns associated with altered spatial and social contexts. A key strength of this study was the individual‐based identification framework, which enabled consistent linkage of data, where similar approaches could be applied in other fenced areas and is encouraged for similar future studies.

### 4.1 Management Implications

The findings from this study demonstrate that expanding available space is an effective strategy for alleviating concentrated browsing pressure and improving elephant well‐being. Considering the continental decline in savanna elephants (Edwards et al. 2024), the issue can be reframed from a surplus of elephants to a lack of available space. By enabling elephants to move more naturally across a larger landscape, vegetation can recover, and elephants can experience increased social interactions, reduced chronic and behavioural stress, and exhibit more natural activity budget profiles. While climate variability strongly influences vegetation dynamics, the absence of increased vegetation degradation following fence removal suggests that expanding functional space for elephants is unlikely to compromise vegetation conditions and may enhance adaptive ecosystem responses (Coetsee et al. 2023). Together, these findings highlight the importance of landscape connectivity as a management strategy for mitigating the ecological and physiological costs associated with spatially restricted elephant populations.

For regions with the capacity to remove internal barriers or expand access, fence removal offers an ecologically sound alternative to more direct population management interventions that vary in levels of intensity and disturbance, such as culling, translocation, or fertility control (Shannon et al. 2013; Giliba et al. 2023). These findings align with broader calls for landscape‐level solutions that enhance ecological resilience and support both elephant populations and biodiversity and should be regarded as the most beneficial management solution to elephant population control (Garaï et al. 2004, 2022; van de Water et al. 2024). Connectivity also enhances tourism value by promoting natural behaviours, increasing social interactions, reducing stress and elephant crowding and thereby reducing associated negative interactions. When paired with thoughtful visitor management, distance regulations, limits on tourism vehicles and guide training, fence removal supports both well‐being and economic sustainability (Szott et al. 2020a). Finally, enhanced connectivity beyond intra‐reserve changes necessitates strengthened collaboration with landowners and conservation areas to anticipate and manage potential cross‐boundary movement. Coordinated landscape planning and conflict mitigation will become increasingly important as barriers are removed and elephants gain greater freedom to move across ecological and administrative boundaries.

## Author Contributions

**Brooke Friswold:** conceptualization (equal), data curation (lead), formal analysis (lead), funding acquisition (lead), investigation (lead), methodology (equal), project administration (lead), resources (lead), software (lead), supervision (equal), validation (lead), visualization (lead), writing – original draft (lead), writing – review and editing (lead). **Antoinette van de Water:** conceptualization (equal), data curation (supporting), funding acquisition (equal), investigation (supporting), methodology (equal), project administration (supporting), resources (equal), supervision (equal), validation (supporting), writing – original draft (supporting), writing – review and editing (supporting). **Brett Mitchell:** conceptualization (supporting), data curation (supporting), methodology (supporting), project administration (supporting), resources (supporting), supervision (supporting), validation (supporting), visualization (supporting), writing – review and editing (supporting). **Sally House:** conceptualization (supporting), data curation (supporting), formal analysis (supporting), investigation (supporting), methodology (supporting), software (supporting), visualization (supporting), writing – original draft (supporting), writing – review and editing (supporting). **Jaco Mitchell:** conceptualization (supporting), data curation (supporting), project administration (supporting), writing – review and editing (supporting). **Harin Aiyanna:** formal analysis (supporting), methodology (supporting), software (supporting), visualization (supporting), writing – original draft (supporting), writing – review and editing (supporting). **Audrey Delsink:** resources (supporting), writing – review and editing (supporting). **Marion Garaï:** supervision (supporting), validation (supporting), writing – review and editing (supporting). **Andre Ganswindt:** data curation (supporting), resources (supporting), writing – review and editing (supporting). **George Gale:** conceptualization (supporting), methodology (supporting), resources (supporting), supervision (lead), validation (supporting), writing – original draft (supporting), writing – review and editing (supporting).

## Funding

This work was supported by Abrivoordieren. Bring The Elephant Home. Petchra Pra Jom Klao Scholarship KMUTT.

## Conflicts of Interest

The authors declare no conflicts of interest.

## Supporting information


**Figure S1:** Seasonal movements of H2; Half Moon from KW, pre‐ (left panel) and post‐ (right panel) fence removal. Each panel illustrates GPS movement data categorised by season (autumn = yellow; winter = brown; spring = light blue; summer = teal). Dashed yellow lines indicate the location of removed internal fences; black lines delineate reserve borders.
**Table S1:** Distribution of faecal samples across explanatory variables used in fGCM analyses (Figure 4).

**Appendix S1:** Ethogram of behaviours, behaviour categories and definitions. Behaviours were classified into categories to support analysis of both activity budgets (using continuous behaviours that are duration based) and behavioural responses (using all‐occurrence behaviours that are count based). The following behaviour categories are used for both types of behaviour (excluding ‘Relaxed’ which was used only for continuous behaviour sampling). *Vigilance* = Behaviours indicating heightened alertness and environmental monitoring, including head, ear, tail, or trunk positioning associated with scanning for potential threats or stimuli. *Disturbance‐related* = Behaviours reflecting avoidance, withdrawal, or acute agitated and arousal responses to perceived disruption or threat. *Affiliative* = Positive or neutral social behaviours that promote social bonding and cohesion, including physical contact, nursing, play sparring and reproductive or courtship interactions. *Passive aggressive* = Low‐intensity dominance or agonistic behaviours that do not involve direct physical conflict, but indicate that physical aggression could occur. *Active aggressive* = High‐intensity agonistic behaviours involving overt conflict, physical engagement, or rapid pursuit, including behaviours intended to dominate, displace, or harm. *Relaxed (continuous only)* = Low‐arousal behaviours associated with routine maintenance, comfort, or inactivity.

## Data Availability

All data underlying the analyses presented in this manuscript are included as Supporting Information for review. A DOI and access link will be provided in the final version of the manuscript.
