# Supplementary material for: Fence Removal Enhances Elephant Movement and Promotes Behavioural, Physiological and Ecological Functioning
Source: Ecol Evol. 2026 May 12;16(5):e73619. doi: 10.1002/ece3.73619 (PMC13163146; doi:10.1002/ece3.73619)
Supplement: Supplementary file 1 — Figure S1: Seasonal movements of H2; Half Moon from KW, pre‐ (left panel) and post‐ (right panel) fence removal. Each panel illustrates GPS movement data categorised by season (autumn = yellow; winter = brown; spring = light blue; summer = teal). Dashed yellow lines indicate the location of removed internal fences; black lines delineate reserve borders. Table S1: Distribution of faecal samples across explanatory variables used in fGCM analyses (Figure 4). [file ECE3-16-e73619-s002.docx]

**Supplementary Materials**


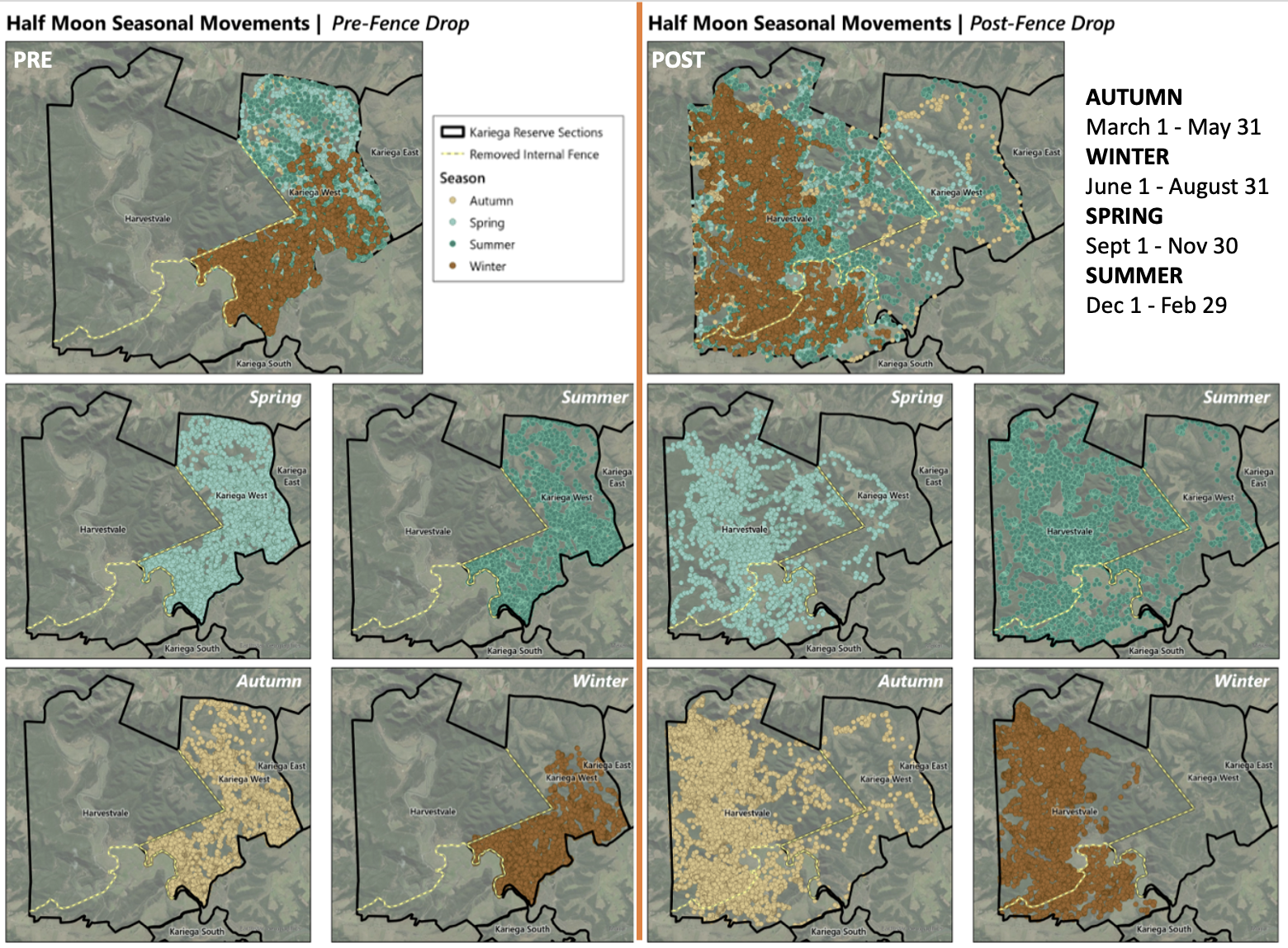


**Figure S1:** Seasonal movements of H2; Half Moon from KW, pre- (left panel) and post- (right panel) fence removal. Each panel illustrates GPS movement data categorised by season (autumn = yellow; winter = brown; spring = light blue; summer = teal). Dashed yellow lines indicate the location of removed internal fences; black lines delineate reserve borders.

**Table S1:** Distribution of faecal samples across explanatory variables used in fGCM analyses (Figure 4).

| **Variable** | **Category** | **Pre (n)** | **Post (n)** |
| --- | --- | --- | --- |
| **Fence status** | Total | 91 | 73 |
| **Seasonal precipitation** | Dry | 53 | 26 |
|  | Wet | 38 | 47 |
| **Sex** | Female | 42 | 29 |
|  | Male | 49 | 44 |
| **Age class** | Adult (A) | 48 | 25 |
|  | Young adult (YA) | 11 | 29 |
|  | Subadult (SA) | 31 | 20 |
